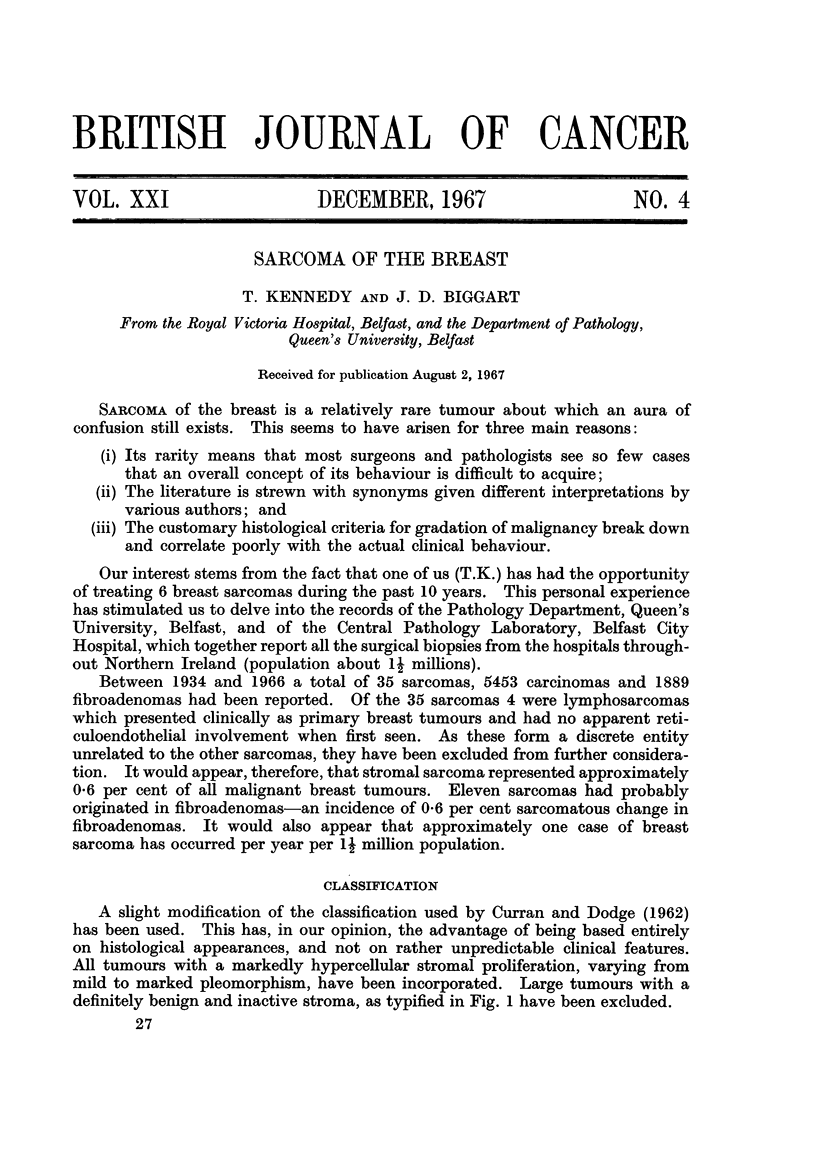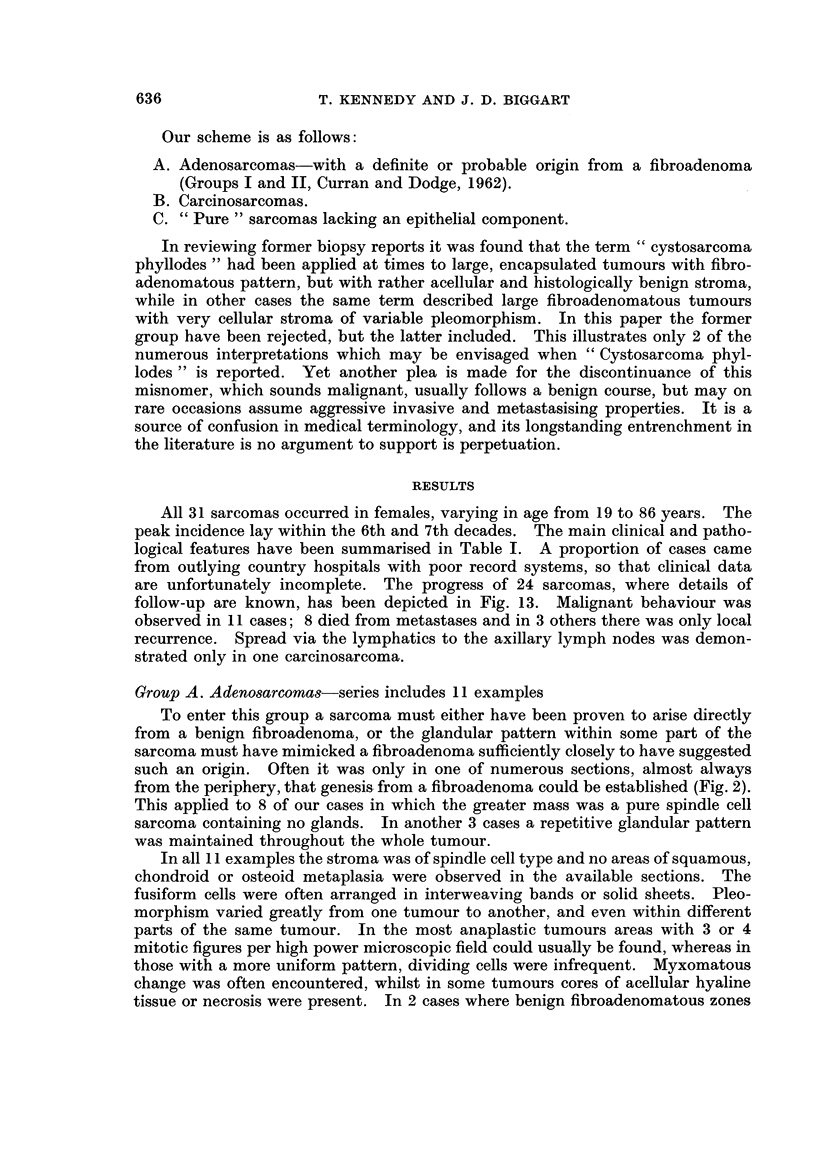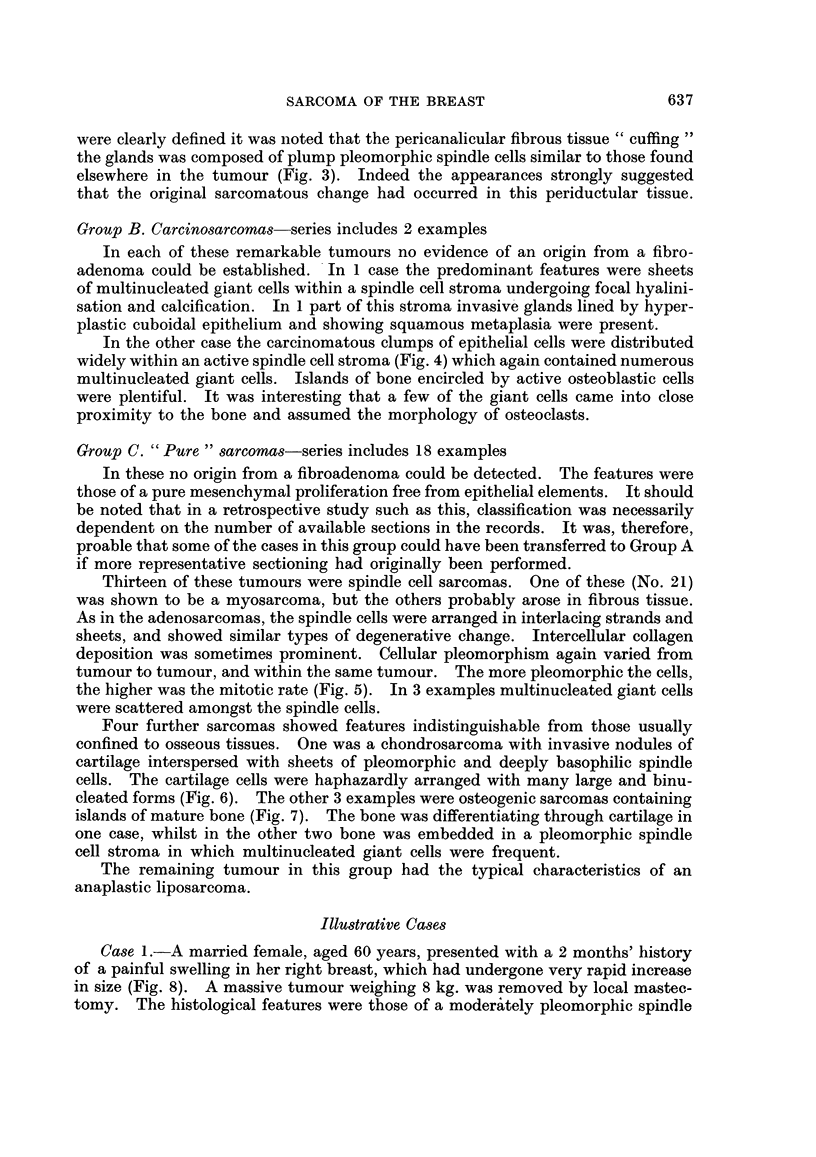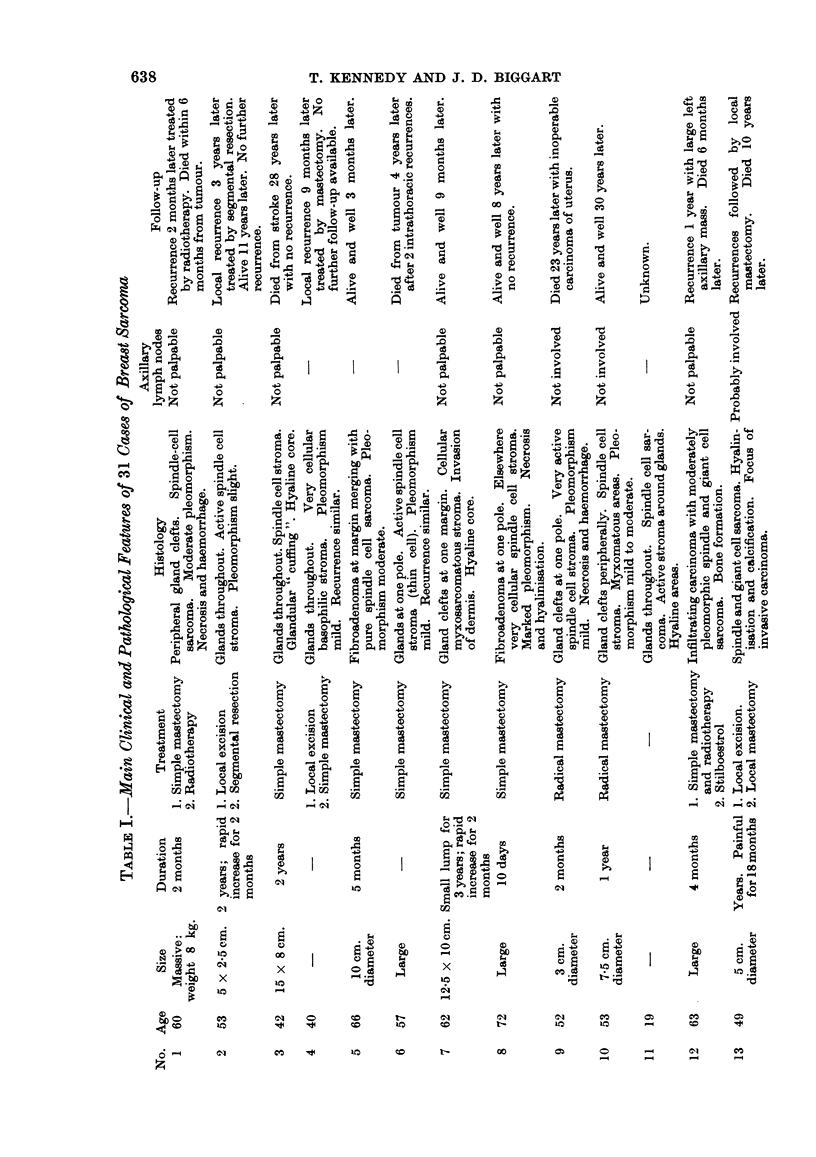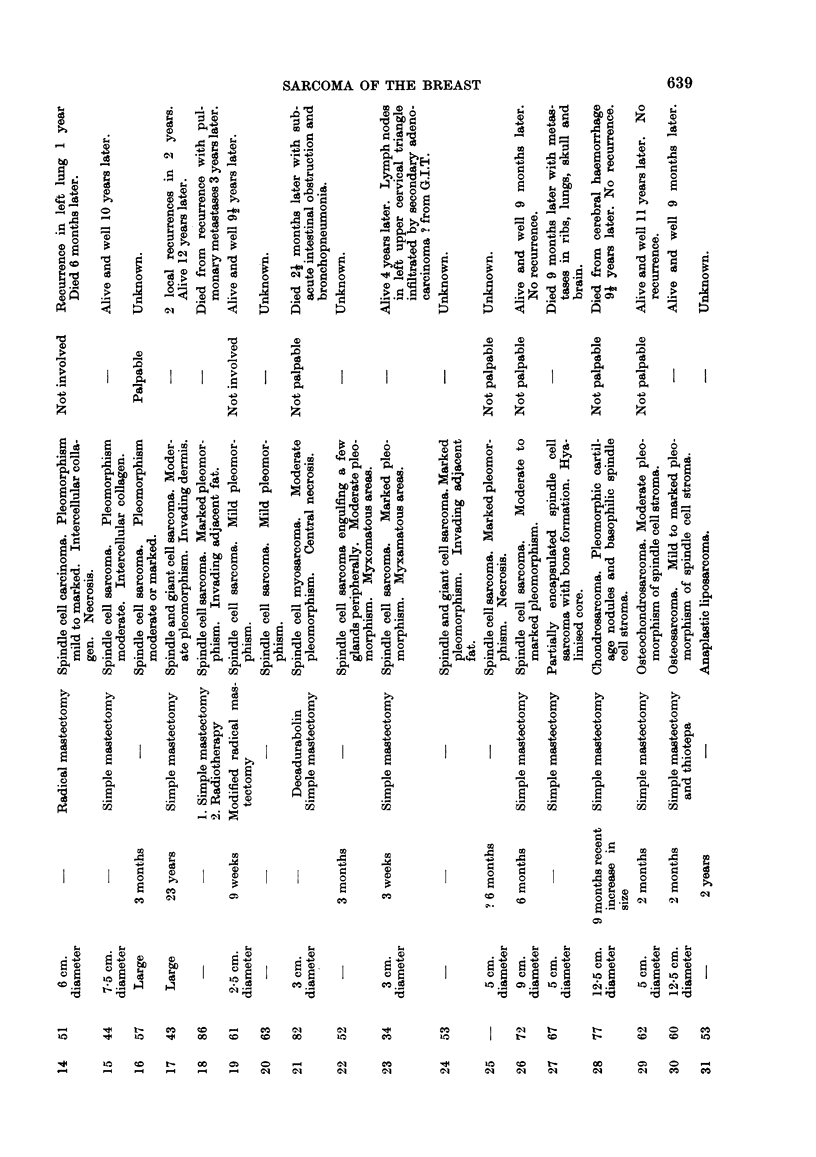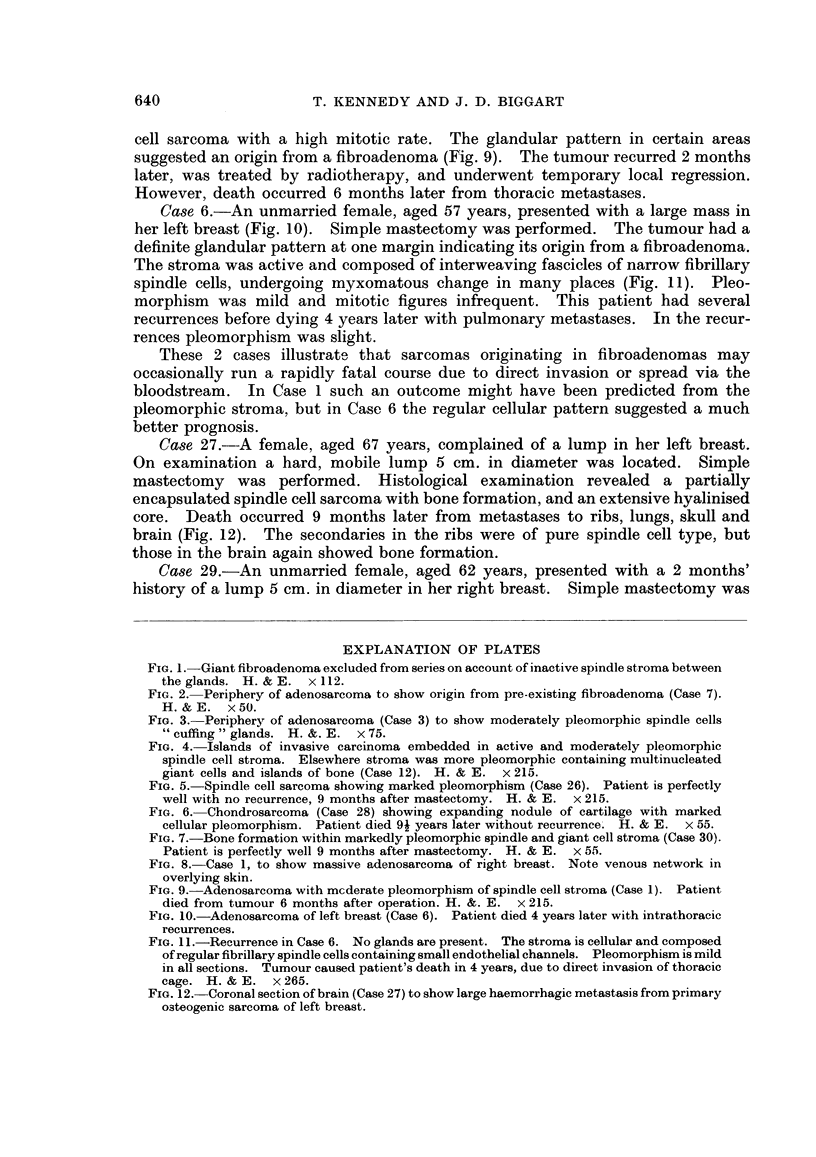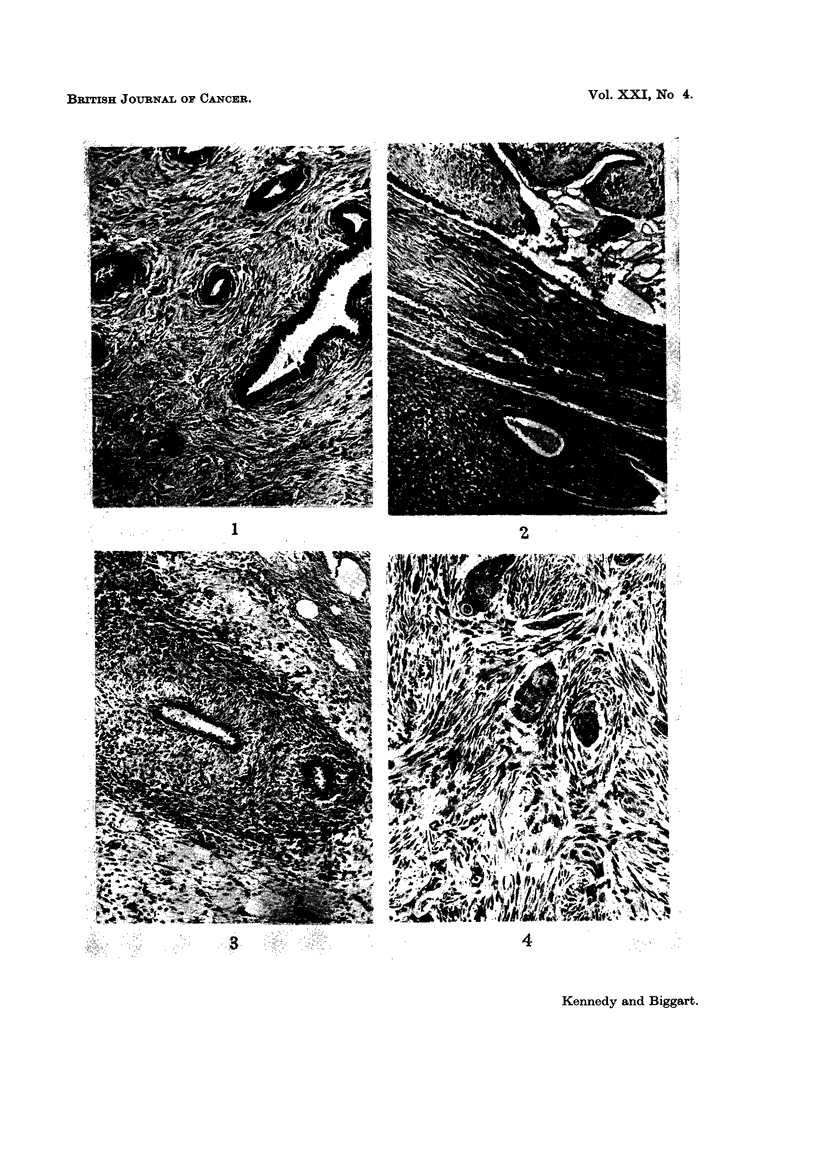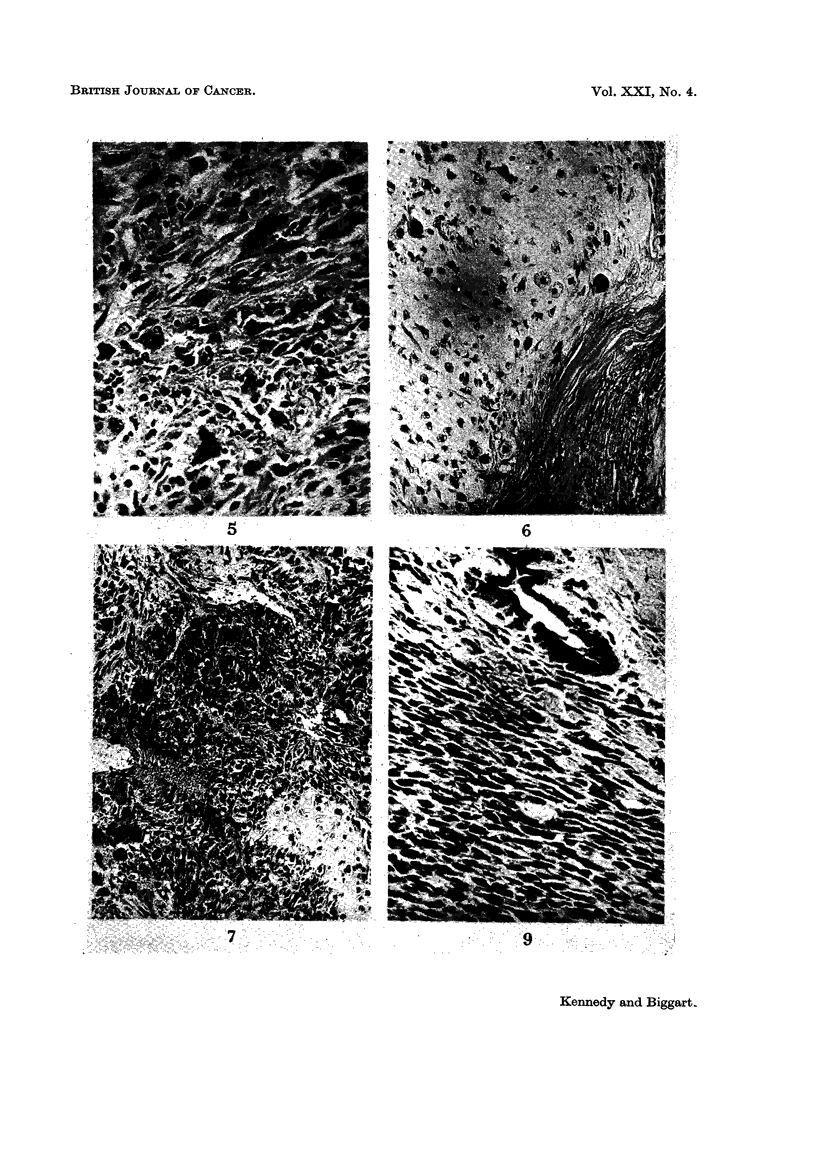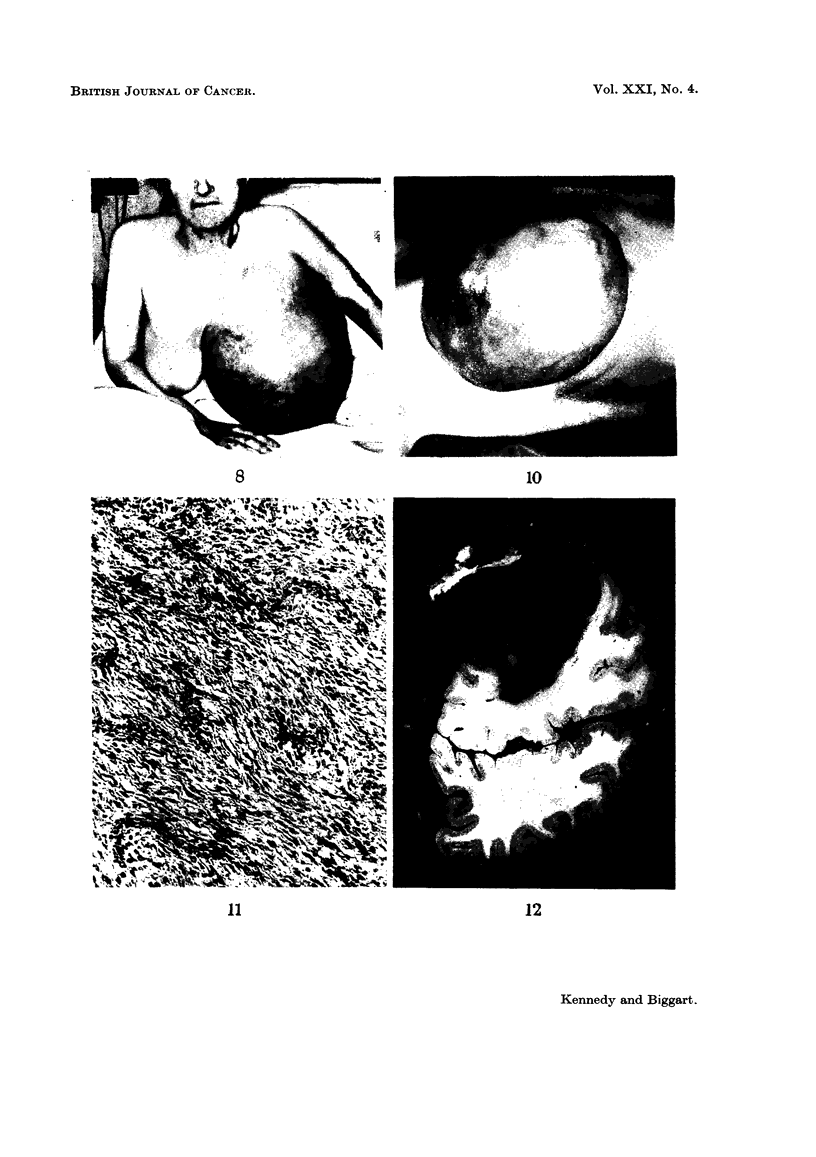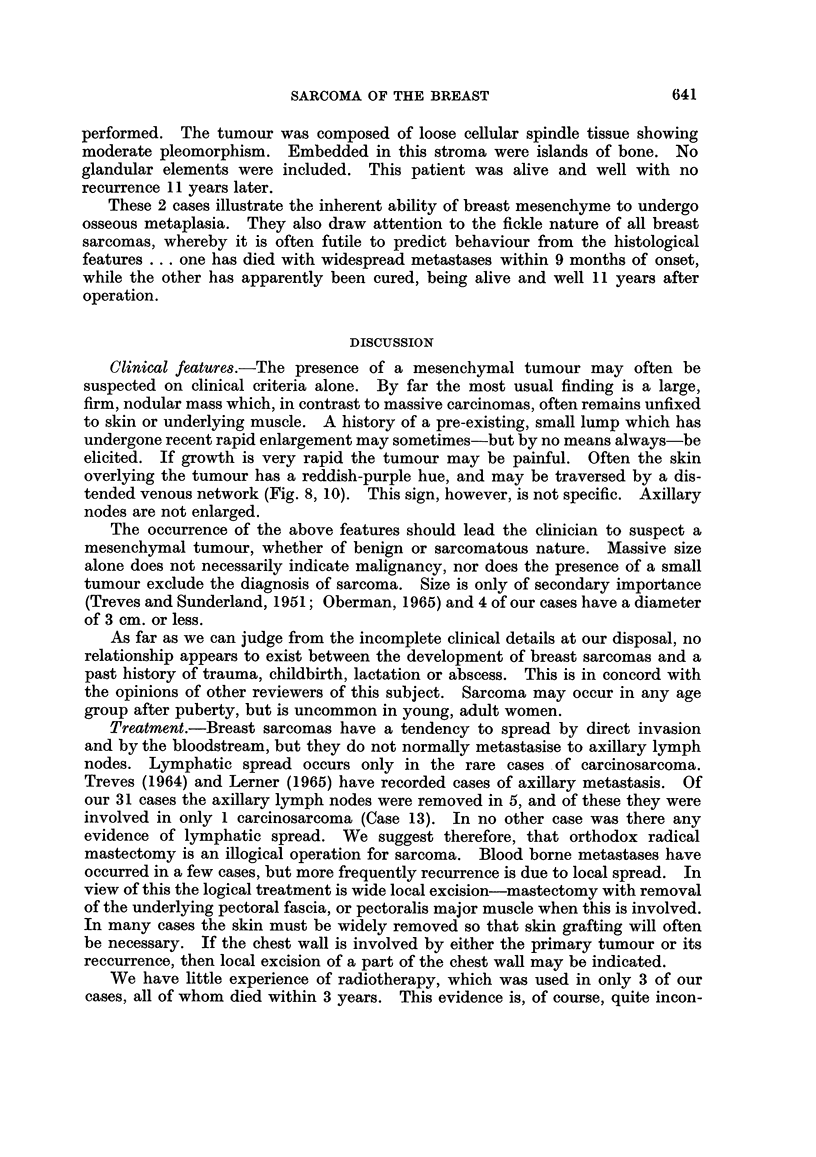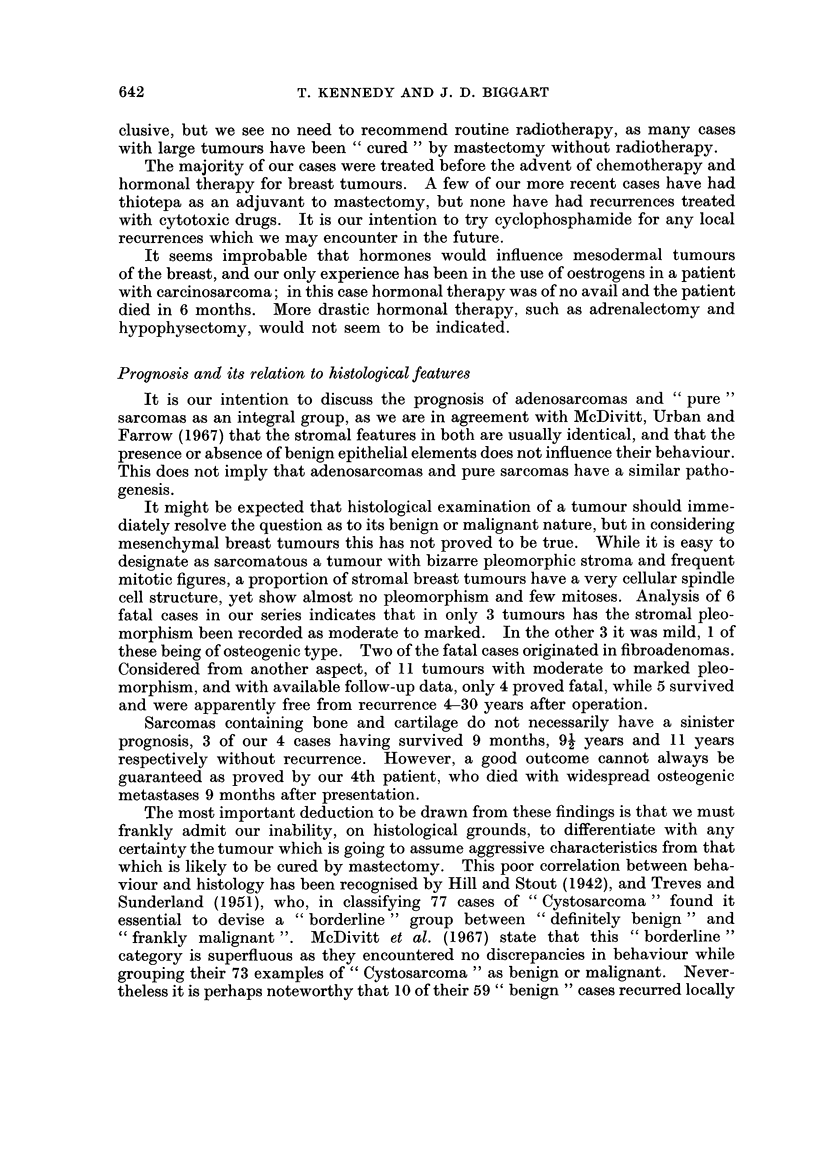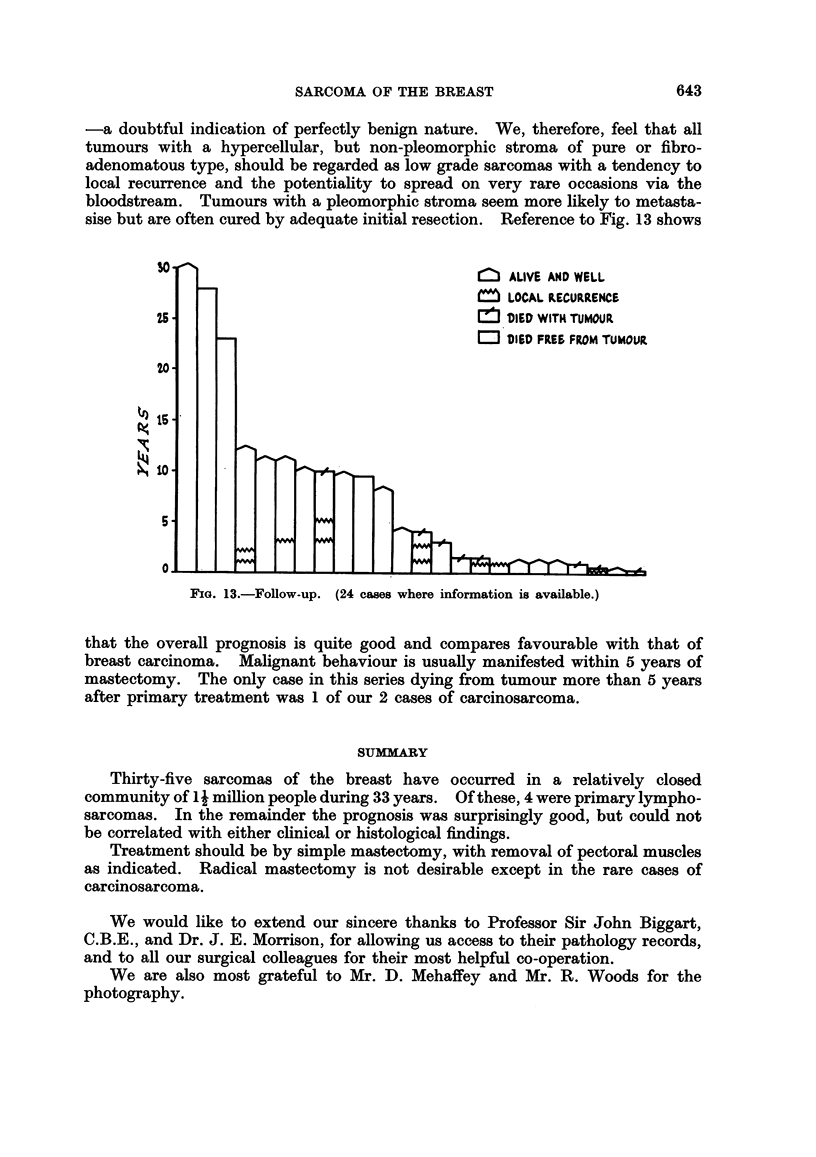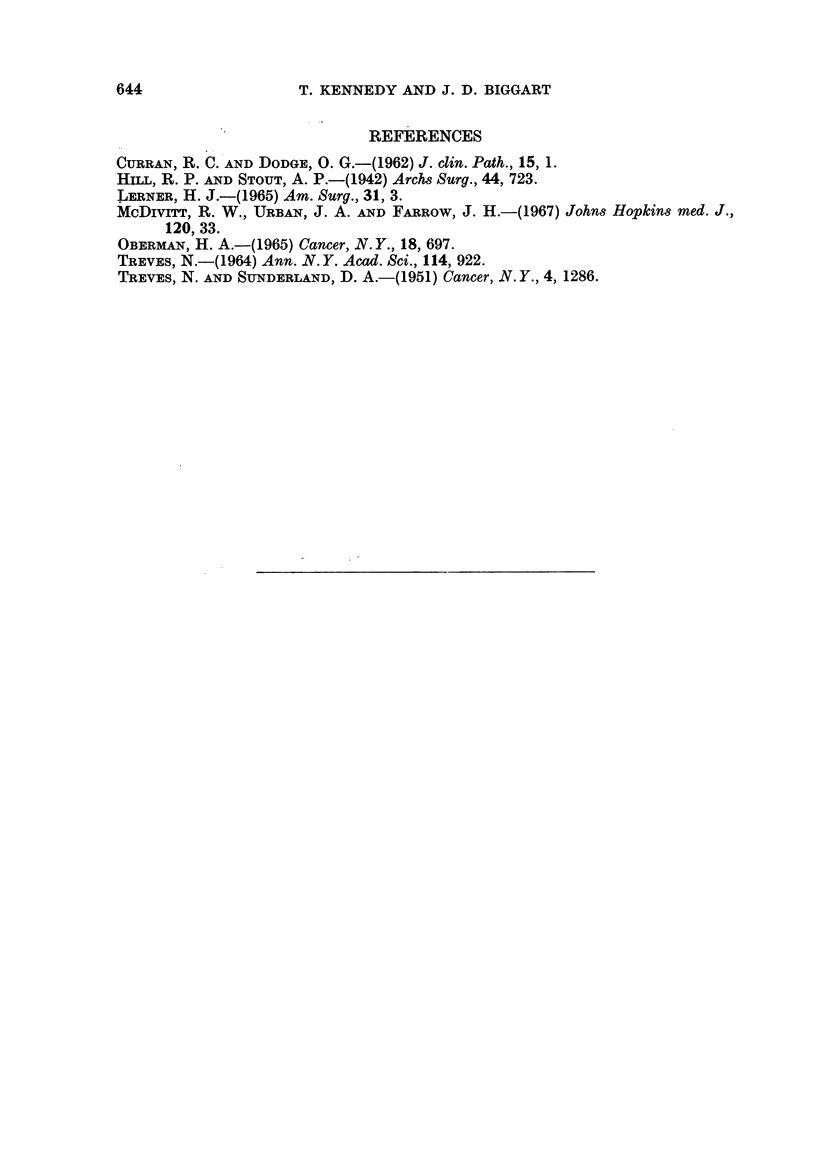# Sarcoma of the breast.

**DOI:** 10.1038/bjc.1967.74

**Published:** 1967-12

**Authors:** T. Kennedy, J. D. Biggart

## Abstract

**Images:**


### BRITISH JOURNAL OF CANCER

VOL. XXI DECEMBER, 1967 NO. 4

### SARCOMA OF THE BREAST

T. KENNEDY AND J. D. BIGGART

*From the Royal Victoria Hospital, Belfast, and the Department of Pathology, Queen's University, Belfast*

Received for publication August 2, 1967

SARCOMA of the breast is a relatively rare tumour about which an aura of confusion still exists. This seems to have arisen for three main reasons:

(i) Its rarity means that most surgeons and pathologists see so few cases that an overall concept of its behaviour is difficult to acquire;
(ii) The literature is strewn with synonyms given different interpretations by various authors; and
(iii) The customary histological criteria for gradation of malignancy break down and correlate poorly with the actual clinical behaviour.

Our interest stems from the fact that one of us (T.K.) has had the opportunity of treating 6 breast sarcomas during the past 10 years. This personal experience has stimulated us to delve into the records of the Pathology Department, Queen's University, Belfast, and of the Central Pathology Laboratory, Belfast City Hospital, which together report all the surgical biopsies from the hospitals throughout Northern Ireland (population about $1\frac{1}{2}$ millions).

Between 1934 and 1966 a total of 35 sarcomas, 5453 carcinomas and 1889 fibroadenomas had been reported. Of the 35 sarcomas 4 were lymphosarcomas which presented clinically as primary breast tumours and had no apparent reticuloendothelial involvement when first seen. As these form a discrete entity unrelated to the other sarcomas, they have been excluded from further consideration. It would appear, therefore, that stromal sarcoma represented approximately 0·6 per cent of all malignant breast tumours. Eleven sarcomas had probably originated in fibroadenomas—an incidence of 0·6 per cent sarcomatous change in fibroadenomas. It would also appear that approximately one case of breast sarcoma has occurred per year per $1\frac{1}{2}$ million population.

### CLASSIFICATION

A slight modification of the classification used by Curran and Dodge (1962) has been used. This has, in our opinion, the advantage of being based entirely on histological appearances, and not on rather unpredictable clinical features. All tumours with a markedly hypercellular stromal proliferation, varying from mild to marked pleomorphism, have been incorporated. Large tumours with a definitely benign and inactive stroma, as typified in Fig. 1 have been excluded.

Our scheme is as follows:

A. Adenosarcomas—with a definite or probable origin from a fibroadenoma (Groups I and II, Curran and Dodge, 1962).
B. Carcinosarcomas.
C. " Pure " sarcomas lacking an epithelial component.

In reviewing former biopsy reports it was found that the term " cystosarcoma phyllodes " had been applied at times to large, encapsulated tumours with fibroadenomatous pattern, but with rather acellular and histologically benign stroma, while in other cases the same term described large fibroadenomatous tumours with very cellular stroma of variable pleomorphism. In this paper the former group have been rejected, but the latter included. This illustrates only 2 of the numerous interpretations which may be envisaged when " Cystosarcoma phyllodes " is reported. Yet another plea is made for the discontinuance of this misnomer, which sounds malignant, usually follows a benign course, but may on rare occasions assume aggressive invasive and metastasising properties. It is a source of confusion in medical terminology, and its longstanding entrenchment in the literature is no argument to support is perpetuation.

### RESULTS

All 31 sarcomas occurred in females, varying in age from 19 to 86 years. The peak incidence lay within the 6th and 7th decades. The main clinical and pathological features have been summarised in Table I. A proportion of cases came from outlying country hospitals with poor record systems, so that clinical data are unfortunately incomplete. The progress of 24 sarcomas, where details of follow-up are known, has been depicted in Fig. 13. Malignant behaviour was observed in 11 cases; 8 died from metastases and in 3 others there was only local recurrence. Spread via the lymphatics to the axillary lymph nodes was demonstrated only in one carcinosarcoma.

*Group A. Adenosarcomas*—series includes 11 examples

To enter this group a sarcoma must either have been proven to arise directly from a benign fibroadenoma, or the glandular pattern within some part of the sarcoma must have mimicked a fibroadenoma sufficiently closely to have suggested such an origin. Often it was only in one of numerous sections, almost always from the periphery, that genesis from a fibroadenoma could be established (Fig. 2). This applied to 8 of our cases in which the greater mass was a pure spindle cell sarcoma containing no glands. In another 3 cases a repetitive glandular pattern was maintained throughout the whole tumour.

In all 11 examples the stroma was of spindle cell type and no areas of squamous, chondroid or osteoid metaplasia were observed in the available sections. The fusiform cells were often arranged in interweaving bands or solid sheets. Pleomorphism varied greatly from one tumour to another, and even within different parts of the same tumour. In the most anaplastic tumours areas with 3 or 4 mitotic figures per high power microscopic field could usually be found, whereas in those with a more uniform pattern, dividing cells were infrequent. Myxomatous change was often encountered, whilst in some tumours cores of acellular hyaline tissue or necrosis were present. In 2 cases where benign fibroadenomatous zones were clearly defined it was noted that the pericanalicular fibrous tissue " cuffing " the glands was composed of plump pleomorphic spindle cells similar to those found elsewhere in the tumour (Fig. 3). Indeed the appearances strongly suggested that the original sarcomatous change had occurred in this periductular tissue.

*Group B. Carcinosarcomas*—series includes 2 examples

In each of these remarkable tumours no evidence of an origin from a fibroadenoma could be established. In 1 case the predominant features were sheets of multinucleated giant cells within a spindle cell stroma undergoing focal hyalinisation and calcification. In 1 part of this stroma invasive glands lined by hyperplastic cuboidal epithelium and showing squamous metaplasia were present.

In the other case the carcinomatous clumps of epithelial cells were distributed widely within an active spindle cell stroma (Fig. 4) which again contained numerous multinucleated giant cells. Islands of bone encircled by active osteoblastic cells were plentiful. It was interesting that a few of the giant cells came into close proximity to the bone and assumed the morphology of osteoclasts.

*Group C. " Pure " sarcomas*—series includes 18 examples

In these no origin from a fibroadenoma could be detected. The features were those of a pure mesenchymal proliferation free from epithelial elements. It should be noted that in a retrospective study such as this, classification was necessarily dependent on the number of available sections in the records. It was, therefore, proable that some of the cases in this group could have been transferred to Group A if more representative sectioning had originally been performed.

Thirteen of these tumours were spindle cell sarcomas. One of these (No. 21) was shown to be a myosarcoma, but the others probably arose in fibrous tissue. As in the adenosarcomas, the spindle cells were arranged in interlacing strands and sheets, and showed similar types of degenerative change. Intercellular collagen deposition was sometimes prominent. Cellular pleomorphism again varied from tumour to tumour, and within the same tumour. The more pleomorphic the cells, the higher was the mitotic rate (Fig. 5). In 3 examples multinucleated giant cells were scattered amongst the spindle cells.

Four further sarcomas showed features indistinguishable from those usually confined to osseous tissues. One was a chondrosarcoma with invasive nodules of cartilage interspersed with sheets of pleomorphic and deeply basophilic spindle cells. The cartilage cells were haphazardly arranged with many large and binucleated forms (Fig. 6). The other 3 examples were osteogenic sarcomas containing islands of mature bone (Fig. 7). The bone was differentiating through cartilage in one case, whilst in the other two bone was embedded in a pleomorphic spindle cell stroma in which multinucleated giant cells were frequent.

The remaining tumour in this group had the typical characteristics of an anaplastic liposarcoma.

### *Illustrative Cases*

*Case* 1.—A married female, aged 60 years, presented with a 2 months' history of a painful swelling in her right breast, which had undergone very rapid increase in size (Fig. 8). A massive tumour weighing 8 kg. was removed by local mastectomy. The histological features were those of a moderately pleomorphic spindle

TABLE I.—*Main Clinical and Pathological Features of* 31 *Cases of Breast Sarcoma*

| No. | Age | Size | Duration | Treatment | Histology | Axillary lymph nodes | Follow-up |
|---|---|---|---|---|---|---|---|
| 1 | 60 | Massive: weight 8 kg. | 2 months | 1. Simple mastectomy<br>2. Radiotherapy | Peripheral gland clefts. Spindle-cell sarcoma. Moderate pleomorphism. Necrosis and haemorrhage. | Not palpable | Recurrence 2 months later treated by radiotherapy. Died within 6 months from tumour. |
| 2 | 53 | 5 × 2·5 cm. | 2 years; rapid increase for 2 months | 1. Local excision<br>2. Segmental resection | Glands throughout. Active spindle cell stroma. Pleomorphism slight. | Not palpable | Local recurrence 3 years later treated by segmental resection. Alive 11 years later. No further recurrence. |
| 3 | 42 | 15 × 8 cm. | 2 years | Simple mastectomy | Glands throughout. Spindle cell stroma. Glandular " cuffing ". Hyaline core. | Not palpable | Died from stroke 28 years later with no recurrence. |
| 4 | 40 | — | — | 1. Local excision<br>2. Simple mastectomy | Glands throughout. Very cellular basophilic stroma. Pleomorphism mild. Recurrence similar. | — | Local recurrence 9 months later treated by mastectomy. No further follow-up available. |
| 5 | 66 | 10 cm. diameter | 5 months | Simple mastectomy | Fibroadenoma at margin merging with pure spindle cell sarcoma. Pleomorphism moderate. | — | Alive and well 3 months later. |
| 6 | 57 | Large | — | Simple mastectomy | Glands at one pole. Active spindle cell stroma (thin cell). Pleomorphism mild. Recurrence similar. | — | Died from tumour 4 years later after 2 intrathoracic recurrences. |
| 7 | 62 | 12·5 × 10 cm. | Small lump for 3 years; rapid increase for 2 months | Simple mastectomy | Gland clefts at one margin. Cellular myxosarcomatous stroma. Invasion of dermis. Hyaline core. | Not palpable | Alive and well 9 months later. |
| 8 | 72 | Large | 10 days | Simple mastectomy | Fibroadenoma at one pole. Elsewhere very cellular spindle cell stroma. Marked pleomorphism. Necrosis and hyalinisation. | Not palpable | Alive and well 8 years later with no recurrence. |
| 9 | 52 | 3 cm. diameter | 2 months | Radical mastectomy | Gland clefts at one pole. Very active spindle cell stroma. Pleomorphism mild. Necrosis and haemorrhage. | Not involved | Died 23 years later with inoperable carcinoma of uterus. |
| 10 | 53 | 7·5 cm. diameter | 1 year | Radical mastectomy | Gland clefts peripherally. Spindle cell stroma. Myxomatous areas. Pleomorphism mild to moderate. | Not involved | Alive and well 30 years later. |
| 11 | 19 | — | — | — | Glands throughout. Spindle cell sarcoma. Active stroma around glands. Hyaline areas. | — | Unknown. |
| 12 | 63 | Large | 4 months | 1. Simple mastectomy and radiotherapy<br>2. Stilboestrol | Infiltrating carcinoma with moderately pleomorphic spindle and giant cell sarcoma. Bone formation. | Not palpable | Recurrence 1 year with large left axillary mass. Died 6 months later. |
| 13 | 49 | 5 cm. diameter | Years. Painful for 18 months | 1. Local excision.<br>2. Local mastectomy | Spindle and giant cell sarcoma. Hyalinisation and calcification. Focus of invasive carcinoma. | Probably involved | Recurrences followed by local mastectomy. Died 10 years later. |

| | | | | | | | |
|---|---|---|---|---|---|---|---|
| 14 | 51 | 6 cm. diameter | — | Radical mastectomy | Spindle cell carcinoma. Pleomorphism mild to marked. Intercellular collagen. Necrosis. | Not involved | Recurrence in left lung 1 year Died 6 months later. |
| 15 | 44 | 7·5 cm. diameter | — | Simple mastectomy | Spindle cell sarcoma. Pleomorphism moderate. Intercellular collagen. | — | Alive and well 10 years later. |
| 16 | 57 | Large | 3 months | — | Spindle cell sarcoma. Pleomorphism moderate or marked. | Palpable | Unknown. |
| 17 | 43 | Large | 23 years | Simple mastectomy | Spindle and giant cell sarcoma. Moderate pleomorphism. Invading dermis. | — | 2 local recurrences in 2 years. Alive 12 years later. |
| 18 | 86 | — | — | 1. Simple mastectomy 2. Radiotherapy | Spindle cell sarcoma. Marked pleomorphism. Invading adjacent fat. | — | Died from recurrence with pulmonary metastases 3 years later. |
| 19 | 61 | 2·5 cm. diameter | 9 weeks | Modified radical mastectomy | Spindle cell sarcoma. Mild pleomorphism. | Not involved | Alive and well 9½ years later. |
| 20 | 63 | — | — | — | Spindle cell sarcoma. Mild pleomorphism. | — | Unknown. |
| 21 | 82 | 3 cm. diameter | — | Decadurabolin Simple mastectomy | Spindle cell myosarcoma. Moderate pleomorphism. Central necrosis. | Not palpable | Died 2½ months later with subacute intestinal obstruction and bronchopneumonia. |
| 22 | 52 | — | 3 months | — | Spindle cell sarcoma engulfing a few glands peripherally. Moderate pleomorphism. Myxomatous areas. | — | Unknown. |
| 23 | 34 | 3 cm. diameter | 3 weeks | Simple mastectomy | Spindle cell sarcoma. Marked pleomorphism. Myxamatous areas. | — | Alive 4 years later. Lymph nodes in left upper cervical triangle infiltrated by secondary adenocarcinoma ? from G.I.T. |
| 24 | 53 | — | — | — | Spindle and giant cell sarcoma. Marked pleomorphism. Invading adjacent fat. | — | Unknown. |
| 25 | — | 5 cm. diameter | ? 6 months | — | Spindle cell sarcoma. Marked pleomorphism. Necrosis. | Not palpable | Unknown. |
| 26 | 72 | 9 cm. diameter | 6 months | Simple mastectomy | Spindle cell sarcoma. Moderate to marked pleomorphism. | Not palpable | Alive and well 9 months later. No recurrence. |
| 27 | 67 | 5 cm. diameter | — | Simple mastectomy | Partially encapsulated spindle cell sarcoma with bone formation. Hyalinised core. | — | Died 9 months later with metastases in ribs, lungs, skull and brain. |
| 28 | 77 | 12·5 cm. diameter | 9 months recent increase in size | Simple mastectomy | Chondrosarcoma. Pleomorphic cartilage nodules and basophilic spindle cell stroma. | Not palpable | Died from cerebral haemorrhage 9½ years later. No recurrence. |
| 29 | 62 | 5 cm. diameter | 2 months | Simple mastectomy | Osteochondrosarcoma. Moderate pleomorphism of spindle cell stroma. | Not palpable | Alive and well 11 years later. No recurrence. |
| 30 | 60 | 12·5 cm. diameter | 2 months | Simple mastectomy and thiotepa | Osteosarcoma. Mild to marked pleomorphism of spindle cell stroma. | — | Alive and well 9 months later. |
| 31 | 53 | — | 2 years | — | Anaplastic liposarcoma. | — | Unknown. |

cell sarcoma with a high mitotic rate. The glandular pattern in certain areas suggested an origin from a fibroadenoma (Fig. 9). The tumour recurred 2 months later, was treated by radiotherapy, and underwent temporary local regression. However, death occurred 6 months later from thoracic metastases.

*Case* 6.—An unmarried female, aged 57 years, presented with a large mass in her left breast (Fig. 10). Simple mastectomy was performed. The tumour had a definite glandular pattern at one margin indicating its origin from a fibroadenoma. The stroma was active and composed of interweaving fascicles of narrow fibrillary spindle cells, undergoing myxomatous change in many places (Fig. 11). Pleomorphism was mild and mitotic figures infrequent. This patient had several recurrences before dying 4 years later with pulmonary metastases. In the recurrences pleomorphism was slight.

These 2 cases illustrate that sarcomas originating in fibroadenomas may occasionally run a rapidly fatal course due to direct invasion or spread via the bloodstream. In Case 1 such an outcome might have been predicted from the pleomorphic stroma, but in Case 6 the regular cellular pattern suggested a much better prognosis.

*Case* 27.—A female, aged 67 years, complained of a lump in her left breast. On examination a hard, mobile lump 5 cm. in diameter was located. Simple mastectomy was performed. Histological examination revealed a partially encapsulated spindle cell sarcoma with bone formation, and an extensive hyalinised core. Death occurred 9 months later from metastases to ribs, lungs, skull and brain (Fig. 12). The secondaries in the ribs were of pure spindle cell type, but those in the brain again showed bone formation.

*Case* 29.—An unmarried female, aged 62 years, presented with a 2 months' history of a lump 5 cm. in diameter in her right breast. Simple mastectomy was

---

EXPLANATION OF PLATES

Fig. 1.—Giant fibroadenoma excluded from series on account of inactive spindle stroma between the glands. H. & E. ×112.

Fig. 2.—Periphery of adenosarcoma to show origin from pre-existing fibroadenoma (Case 7). H. & E. ×50.

Fig. 3.—Periphery of adenosarcoma (Case 3) to show moderately pleomorphic spindle cells "cuffing" glands. H. &. E. ×75.

Fig. 4.—Islands of invasive carcinoma embedded in active and moderately pleomorphic spindle cell stroma. Elsewhere stroma was more pleomorphic containing multinucleated giant cells and islands of bone (Case 12). H. & E. ×215.

Fig. 5.—Spindle cell sarcoma showing marked pleomorphism (Case 26). Patient is perfectly well with no recurrence, 9 months after mastectomy. H. & E. ×215.

Fig. 6.—Chondrosarcoma (Case 28) showing expanding nodule of cartilage with marked cellular pleomorphism. Patient died 9½ years later without recurrence. H. & E. ×55.

Fig. 7.—Bone formation within markedly pleomorphic spindle and giant cell stroma (Case 30). Patient is perfectly well 9 months after mastectomy. H. & E. ×55.

Fig. 8.—Case 1, to show massive adenosarcoma of right breast. Note venous network in overlying skin.

Fig. 9.—Adenosarcoma with moderate pleomorphism of spindle cell stroma (Case 1). Patient died from tumour 6 months after operation. H. &. E. ×215.

Fig. 10.—Adenosarcoma of left breast (Case 6). Patient died 4 years later with intrathoracic recurrences.

Fig. 11.—Recurrence in Case 6. No glands are present. The stroma is cellular and composed of regular fibrillary spindle cells containing small endothelial channels. Pleomorphism is mild in all sections. Tumour caused patient's death in 4 years, due to direct invasion of thoracic cage. H. & E. ×265.

Fig. 12.—Coronal section of brain (Case 27) to show large haemorrhagic metastasis from primary osteogenic sarcoma of left breast.

1

2

3

4

Kennedy and Biggart.

5

6

7

9

Kennedy and Biggart.

8

10

11

12

performed. The tumour was composed of loose cellular spindle tissue showing moderate pleomorphism. Embedded in this stroma were islands of bone. No glandular elements were included. This patient was alive and well with no recurrence 11 years later.

These 2 cases illustrate the inherent ability of breast mesenchyme to undergo osseous metaplasia. They also draw attention to the fickle nature of all breast sarcomas, whereby it is often futile to predict behaviour from the histological features . . . one has died with widespread metastases within 9 months of onset, while the other has apparently been cured, being alive and well 11 years after operation.

### DISCUSSION

*Clinical features.*—The presence of a mesenchymal tumour may often be suspected on clinical criteria alone. By far the most usual finding is a large, firm, nodular mass which, in contrast to massive carcinomas, often remains unfixed to skin or underlying muscle. A history of a pre-existing, small lump which has undergone recent rapid enlargement may sometimes—but by no means always—be elicited. If growth is very rapid the tumour may be painful. Often the skin overlying the tumour has a reddish-purple hue, and may be traversed by a distended venous network (Fig. 8, 10). This sign, however, is not specific. Axillary nodes are not enlarged.

The occurrence of the above features should lead the clinician to suspect a mesenchymal tumour, whether of benign or sarcomatous nature. Massive size alone does not necessarily indicate malignancy, nor does the presence of a small tumour exclude the diagnosis of sarcoma. Size is only of secondary importance (Treves and Sunderland, 1951; Oberman, 1965) and 4 of our cases have a diameter of 3 cm. or less.

As far as we can judge from the incomplete clinical details at our disposal, no relationship appears to exist between the development of breast sarcomas and a past history of trauma, childbirth, lactation or abscess. This is in concord with the opinions of other reviewers of this subject. Sarcoma may occur in any age group after puberty, but is uncommon in young, adult women.

*Treatment.*—Breast sarcomas have a tendency to spread by direct invasion and by the bloodstream, but they do not normally metastasise to axillary lymph nodes. Lymphatic spread occurs only in the rare cases of carcinosarcoma. Treves (1964) and Lerner (1965) have recorded cases of axillary metastasis. Of our 31 cases the axillary lymph nodes were removed in 5, and of these they were involved in only 1 carcinosarcoma (Case 13). In no other case was there any evidence of lymphatic spread. We suggest therefore, that orthodox radical mastectomy is an illogical operation for sarcoma. Blood borne metastases have occurred in a few cases, but more frequently recurrence is due to local spread. In view of this the logical treatment is wide local excision—mastectomy with removal of the underlying pectoral fascia, or pectoralis major muscle when this is involved. In many cases the skin must be widely removed so that skin grafting will often be necessary. If the chest wall is involved by either the primary tumour or its reccurrence, then local excision of a part of the chest wall may be indicated.

We have little experience of radiotherapy, which was used in only 3 of our cases, all of whom died within 3 years. This evidence is, of course, quite inconclusive, but we see no need to recommend routine radiotherapy, as many cases with large tumours have been " cured " by mastectomy without radiotherapy.

The majority of our cases were treated before the advent of chemotherapy and hormonal therapy for breast tumours. A few of our more recent cases have had thiotepa as an adjuvant to mastectomy, but none have had recurrences treated with cytotoxic drugs. It is our intention to try cyclophosphamide for any local recurrences which we may encounter in the future.

It seems improbable that hormones would influence mesodermal tumours of the breast, and our only experience has been in the use of oestrogens in a patient with carcinosarcoma; in this case hormonal therapy was of no avail and the patient died in 6 months. More drastic hormonal therapy, such as adrenalectomy and hypophysectomy, would not seem to be indicated.

*Prognosis and its relation to histological features*

It is our intention to discuss the prognosis of adenosarcomas and " pure " sarcomas as an integral group, as we are in agreement with McDivitt, Urban and Farrow (1967) that the stromal features in both are usually identical, and that the presence or absence of benign epithelial elements does not influence their behaviour. This does not imply that adenosarcomas and pure sarcomas have a similar pathogenesis.

It might be expected that histological examination of a tumour should immediately resolve the question as to its benign or malignant nature, but in considering mesenchymal breast tumours this has not proved to be true. While it is easy to designate as sarcomatous a tumour with bizarre pleomorphic stroma and frequent mitotic figures, a proportion of stromal breast tumours have a very cellular spindle cell structure, yet show almost no pleomorphism and few mitoses. Analysis of 6 fatal cases in our series indicates that in only 3 tumours has the stromal pleomorphism been recorded as moderate to marked. In the other 3 it was mild, 1 of these being of osteogenic type. Two of the fatal cases originated in fibroadenomas. Considered from another aspect, of 11 tumours with moderate to marked pleomorphism, and with available follow-up data, only 4 proved fatal, while 5 survived and were apparently free from recurrence 4–30 years after operation.

Sarcomas containing bone and cartilage do not necessarily have a sinister prognosis, 3 of our 4 cases having survived 9 months, 9½ years and 11 years respectively without recurrence. However, a good outcome cannot always be guaranteed as proved by our 4th patient, who died with widespread osteogenic metastases 9 months after presentation.

The most important deduction to be drawn from these findings is that we must frankly admit our inability, on histological grounds, to differentiate with any certainty the tumour which is going to assume aggressive characteristics from that which is likely to be cured by mastectomy. This poor correlation between behaviour and histology has been recognised by Hill and Stout (1942), and Treves and Sunderland (1951), who, in classifying 77 cases of " Cystosarcoma " found it essential to devise a " borderline " group between " definitely benign " and " frankly malignant ". McDivitt *et al.* (1967) state that this " borderline " category is superfluous as they encountered no discrepancies in behaviour while grouping their 73 examples of " Cystosarcoma " as benign or malignant. Nevertheless it is perhaps noteworthy that 10 of their 59 " benign " cases recurred locally

—a doubtful indication of perfectly benign nature. We, therefore, feel that all tumours with a hypercellular, but non-pleomorphic stroma of pure or fibro-adenomatous type, should be regarded as low grade sarcomas with a tendency to local recurrence and the potentiality to spread on very rare occasions via the bloodstream. Tumours with a pleomorphic stroma seem more likely to metastasise but are often cured by adequate initial resection. Reference to Fig. 13 shows

FIG. 13.—Follow-up. (24 cases where information is available.)

that the overall prognosis is quite good and compares favourable with that of breast carcinoma. Malignant behaviour is usually manifested within 5 years of mastectomy. The only case in this series dying from tumour more than 5 years after primary treatment was 1 of our 2 cases of carcinosarcoma.

### SUMMARY

Thirty-five sarcomas of the breast have occurred in a relatively closed community of 1½ million people during 33 years. Of these, 4 were primary lymphosarcomas. In the remainder the prognosis was surprisingly good, but could not be correlated with either clinical or histological findings.

Treatment should be by simple mastectomy, with removal of pectoral muscles as indicated. Radical mastectomy is not desirable except in the rare cases of carcinosarcoma.

We would like to extend our sincere thanks to Professor Sir John Biggart, C.B.E., and Dr. J. E. Morrison, for allowing us access to their pathology records, and to all our surgical colleagues for their most helpful co-operation.

We are also most grateful to Mr. D. Mehaffey and Mr. R. Woods for the photography.

### REFERENCES

Curran, R. C. and Dodge, O. G.—(1962) *J. clin. Path.*, **15**, 1.
Hill, R. P. and Stout, A. P.—(1942) *Archs Surg.*, **44**, 723.
Lerner, H. J.—(1965) *Am. Surg.*, **31**, 3.
McDivitt, R. W., Urban, J. A. and Farrow, J. H.—(1967) *Johns Hopkins med. J.*, **120**, 33.
Oberman, H. A.—(1965) *Cancer, N.Y.*, **18**, 697.
Treves, N.—(1964) *Ann. N.Y. Acad. Sci.*, **114**, 922.
Treves, N. and Sunderland, D. A.—(1951) *Cancer, N.Y.*, **4**, 1286.